# Interleukin-4 Aggravates LPS-Induced Striatal Neurodegeneration In Vivo via Oxidative Stress and Polarization of Microglia/Macrophages

**DOI:** 10.3390/ijms23010571

**Published:** 2022-01-05

**Authors:** Jaegeun Jang, Ahreum Hong, Youngcheul Chung, Byungkwan Jin

**Affiliations:** 1 Department of Neuroscience, Graduate School of Medicine, Kyung Hee University, Seoul 02447, Korea; jesspass@daum.net (J.J.); hongar@khu.ac.kr (A.H.); 2 Department of Predictive Toxicology, Korea Institute of Toxicology, Daejeon 34114, Korea; 3 Department of Biochemistry & Molecular Biology, School of Medicine, Kyung Hee University, Seoul 02447, Korea

**Keywords:** microglia, neurodegeneration, interleukin-4, oxidative/nitrosative stress, microglia/macrophage polarization

## Abstract

The present study investigated the effects of interleukin (IL)-4 on striatal neurons in lipopolysaccharide (LPS)-injected rat striatum in vivo. Either LPS or PBS as a control was unilaterally injected into the striatum, and brain tissues were processed for immunohistochemical and Nissl staining or for hydroethidine histochemistry at the indicated time points after LPS injection. Analysis by NeuN and Nissl immunohistochemical staining showed a significant loss of striatal neurons at 1, 3, and 7 days post LPS. In parallel, IL-4 immunoreactivity was upregulated as early as 1 day, reached a peak at 3 days, and was sustained up to 7 days post LPS. Increased levels of IL-4 immunoreactivity were exclusively detected in microglia/macrophages, but not in neurons nor astrocytes. The neutralizing antibody (NA) for IL-4 significantly protects striatal neurons against LPS-induced neurotoxicity in vivo. Accompanying neuroprotection, IL-4NA inhibited activation of microglia/macrophages, production of reactive oxygen species (ROS), ROS-derived oxidative damage and nitrosative stress, and produced polarization of microglia/macrophages shifted from M1 to M2. These results suggest that endogenous IL-4 expressed in LPS-activated microglia/macrophages contributes to striatal neurodegeneration in which oxidative/nitrosative stress and M1/M2 polarization are implicated.

## 1. Introduction

Oxidative/nitrosative stress is a condition produced by the imbalance between oxidants and antioxidants [1], which results in the generation of excessive levels of reactive oxygen species (ROS)/reactive nitrogen species (RNS) such as superoxide radical anion (O_2_^−^) and nitric oxide (NO) [2]. ROS/RNS can inflict serious damage on cellular biomolecules, including lipids and membranes, proteins, and DNA [3,4]. Accumulating evidence suggest that oxidative/nitrosative stress has been implicated in neurodegenerative diseases, including amyotrophic lateral sclerosis (ALS), Parkinson’s disease (PD), and Alzheimer’s disease [5,6,7], and can be a potential therapeutic target for the treatment of neurodegenerative diseases.

Microglia are the resident macrophages in the brain and are responsible for the maintenance of brain homeostasis and neuroinflammation [8,9,10]. Activated microglia become neurotoxic by generating ROS/RNS [11,12] and are classified as being in the classical activation (M1) or the alternative activation state (M2) [13]. After pathogenic stimuli, M1-like microglia produce proinflammatory molecules, including inducible nitric oxide synthase (iNOS) [14], whereas M2-like microglia remove cellular debris through phagocytosis and release anti-inflammatory molecules, including arginase (Arg1) [15].

Lipopolysaccharide (LPS) is well known as an endotoxin that is found in the outer membrane of gram-negative bacteria. LPS increases ROS/RNS, releases inflammatory cytokines, and induces an immune response, such as microglial activation, as well as microglial polarization [16,17,18]. Administration of LPS to animals induces [19] loss of nigral dopamine neurons [20], hippocampal neurons, and cortical neurons [21]. These studies showed that LPS-induced neurotoxicity was associated with activated microglia-derived ROS/RNS production and/or microglial polarization.

Interleukin-4 (IL-4), a well-known anti-inflammatory cytokine, regulates the polarization of the periphery macrophage phenotype [22,23], and inhibits the production of inflammatory mediators, such as interleukin-1β and tumor necrosis factor-α (TNF) [24,25]. IL-4 is expressed in microglia in the brain and can play both beneficial and detrimental roles [26]. IL-4 exerts neuroprotective effects on neurons in the animal models of experimental autoimmune encephalomyelitis (EAE) [27] and Alzheimer’s disease [28]. By contrast, IL-4 potentiates beta-amyloid- and prothrombin kringle-2 (pKr-2)-induced neurotoxicity by producing ROS/RNS or proinflammatory cytokines [24,29,30]. Although detailed mechanisms for inconsistent actions of IL-4 under neuroinflammatory conditions are uncertain, these results suggest that IL-4 plays a crucial role in neuroinflammation. In this study, we investigated whether IL-4 that is endogenously expressed within activated microglia is associated with neurodegeneration by regulating oxidative/nitrosative stress and microglial polarization in the LPS-induced striatum in vivo.

## 2. Results

### 2.1. Interleukin-4 Contributes to Neurodegeneration and Microglial Activation in LPS-Injected Rat Striatum In Vivo

**Figure 1:**
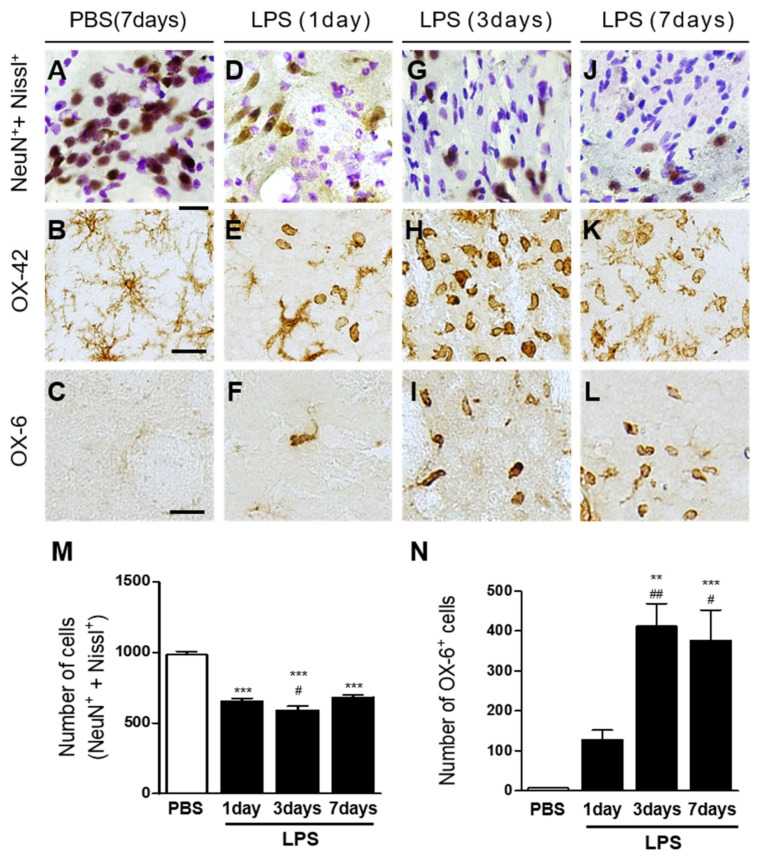
LPS induces neurodegeneration and microglial activation in the rat striatum in vivo. Animals unilaterally received injection of phosphate-buffered saline (PBS) (**A**–**C**) as a control or lipopolysaccharide (LPS; (**D**–**L**); 5 µg/3 µL) into the rat striatum and were transcardially perfused at indicated time points. The brain tissues were processed for Nissl staining (**A**,**D**,**G**,**J**) and immunohistochemical staining with NeuN (neuronal nuclei) and Nissl costaining (**A**,**D**,**G**,**J**) or OX-42 (complement receptor3, CR3; (**B**,**E**,**H**,**K**)) to identify microglia/macrophages, or OX-6 (major histocompatibility complex class Ⅱ; (**C**,**F**,**I**,**L**)) to identify activated microglia at 1 day (**D**–**F**), 3 days (**G**–**I**), and 7 days (**J**–**L**) after intrastriatal injection of LPS. Scale bars, 20 µm (**A**,**D**,**G**,**J**), 25 µm (**B**,**C**,**E**,**F**,**H**,**I**,**K**,**L**). (**M**) Quantification of NeuN^+^ and Nissl^+^ cells in the LPS-injected striatum (Total area = 4.6 × 10^5^ μm^2^). *** *p* < 0.001, significantly different from PBS (control). Data are presented as the mean ± SEM; n of animals = 4 to 5 in each group, ANOVA with Newman–Keuls analysis. (**N**) Quantification of OX-6^+^ cells in the LPS-injected striatum (total area = 4.6 × 10^5^ μm^2^). ** *p* < 0.01, significantly different from PBS (control). *** *p* < 0.001, significantly different from PBS (control). ^##^ *p* < 0.01, significantly different from LPS 1 day. ^#^ *p* < 0.05, significantly different from LPS 1 day. Data are presented as the mean ± SEM; *n* of animals = 4 to 5 in each group, ANOVA with Newman–Keuls analysis.

Animals received a unilateral injection of LPS (5 μg) or PBS as a control into the striatum. Brain sections were processed for Nissl staining and immunohistochemical staining for NeuN to identify general striatal neurons at the indicated time points. Histological examination showed healthy and large Nissl^+^ cells in the striatum at 7 days after PBS injection (Figure 1A). By marked contrast, LPS produced a significant loss of Nissl^+^ cells in the striatum as early as 1 day after LPS injection (Figure 1D), which was maintained up to 3 days (Figure 1G) and 7 days (Figure 1J) after LPS injection. Similar to Nissl staining, NeuN immunostaining showed that PBS injection caused no apparent loss of NeuN^+^ neurons (Figure 1A), while LPS injection significantly reduced NeuN^+^ neurons as early as 1 day after LPS injection (Figure 1D), with the reduction being maintained up to 3 days (Figure 1G) and 7 days (Figure 1J) after LPS injection. When the number of Nissl^+^/NeuN^+^ cells was quantified, LPS was found to significantly attenuate the number of Nissl^+^/NeuN^+^ cells compared with PBS (Figure 1M), indicating substantial loss of striatal neurons.

To determine activation of microglia in the stratum by LPS in vivo, sections adjacent to those used for NeuN immunostaining were processed for immunohistochemical staining using antibodies against OX-42 and OX-6 to detect microglial activation, as recently described [31]. In the PBS-injected striatum, OX-42^+^ cells exhibited the resting state with small cell bodies and ramified processes (Figure 1B). In contrast, the majority of OX-42^+^ cells exhibited the activated state with large cell bodies and short, thick, or no processes, which were observed as early as 1 day after LPS injection (Figure 1E), significantly increased at 3 days after LPS injection (Figure 1H), and were sustained up to 7 days after LPS injection (Figure 1K). Similar to OX-42^+^ cells, OX-6^+^ cells were observed in the striatum as early as 1 day after LPS injection (Figure 1F), significantly increased at 3 days after LPS injection (Figure 1I), and were maintained up to 7 days after LPS injection (Figure 1L), whereas in the PBS-injected control, few of the OX-6^+^ cells were seen (Figure 1C). When the number of OX-6^+^ cells was quantified, LPS was found to significantly increase the number of OX-6^+^ cells in a time-dependent manner, compared with PBS (Figure 1N).

**Figure 2:**
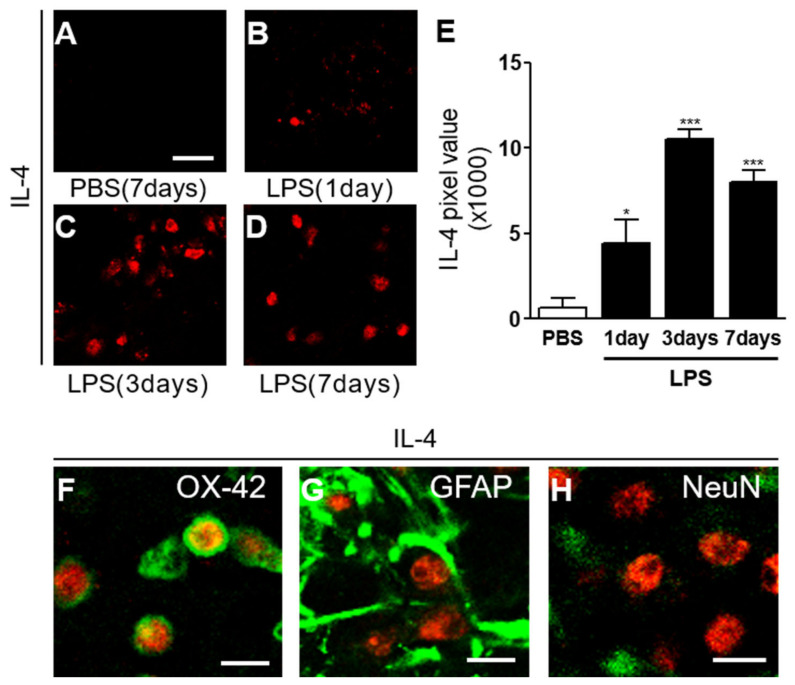
IL-4 expression in LPS-lesioned striatum in vivo. Animals received a unilateral injection of PBS (**A**) or LPS (**B**–**D**) into the rat striatum and were transcardially perfused at various time points. The brain sections were processed for immunohistochemical staining. (**A**–**E**) Immunofluorescence images of IL-4 (**A**–**D**) and quantification (**E**) in the rat striatum at indicated time points. Scale bar, 25 µm. * *p* < 0.05, significantly different from PBS (control). *** *p* < 0.001 significantly different from PBS-injected control. Mean ± SEM; n of animals = 4 to 6 in each group, ANOVA with Bonferroni test. (**F**–**H**) Double immunofluorescence images of IL-4 ((**F**), red) and OX-42 ((**F**), green), or IL-4 ((**G**), red) and GFAP ((**G**), green), or IL-4 ((**H**), red) and NeuN ((**H**), green), and both images are merged (yellow). *n* of animals = 4. Scale bar, 15 µm.

To investigate whether LPS could induce interleukin-4 (IL-4) protein expression in the striatum, sections adjacent to those used for OX-42 immunostaining were processed for immunohistochemical staining using antibodies against IL-4. Immunohistochemical analysis showed no expression of IL-4 in PBS-injected striatum (Figure 2A,E). By marked contrast, LPS produced the expression of IL-4 as early as 1 day post LPS (Figure 2B,E), maximal at 3 days post LPS (Figure 2C,E), and was maintained up to 7 days post LPS (Figure 2D,E). Expression of IL-4 was analyzed in OX-42^+^ microglia, GFAP^+^ astrocytes, and NeuN^+^ neurons at 3 days post LPS. Immunohistochemical analysis revealed expression of IL-4 exclusively in OX-42^+^ microglia (Figure 2F), but not in GFAP^+^ astrocytes (Figure 2G), nor in NeuN^+^ neurons (Figure 2H) in the LPS-injected striatum.

**Figure 3:**
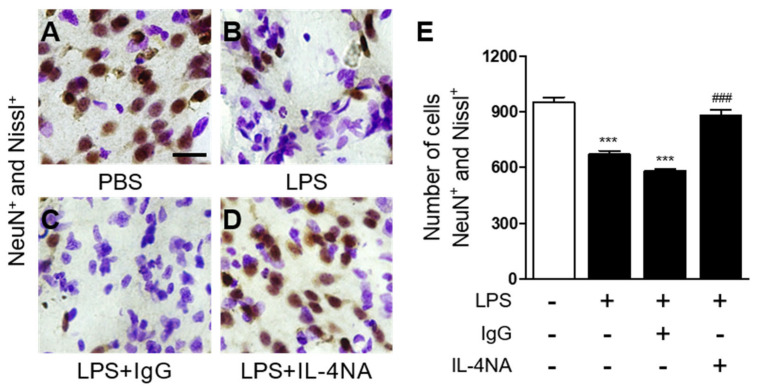
IL-4 causes LPS-induced neurotoxicity in the striatum in vivo. Animals unilaterally received intrastriatal injection of PBS (**A**) as a control, LPS (**B**), LPS+non-specific IgG (1 µg/µL; (**C**)), and LPS+IL-4NA (1 µg/µL; (**D**)). At 7 days after injection, animals were transcardially perfused, and brain tissues were processed for neuronal nuclei (NeuN) immunostaining and Nissl staining at 7 days post LPS injection. Scale bar, 25 µm. (**E**) Quantification of NeuN^+^ and Nissl^+^ cells in the LPS-injected striatum (Total area = 4.6 × 10^5^ μm^2^). *** *p* < 0.001 significantly different from PBS (control). ^###^ *p* < 0.001 significantly different from LPS mean ± SEM; n of animals = 5 to 6 in each group. ANOVA and Bonferroni analysis.

**Figure 4:**
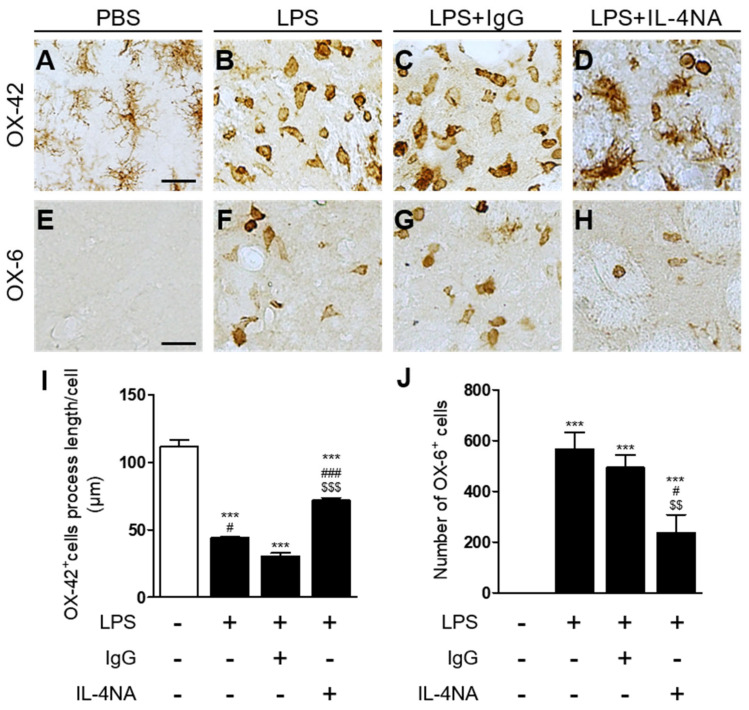
IL-4 contributes to microglial activation in the LPS-injected striatum in vivo. Animals intrastriatally received a unilateral injection of PBS (**A**,**E**) as a control, LPS (**B**,**F**), LPS+non-specific IgG (1 µg/µL; (**C**,**G**)), and LPS+IL-4NA (1 µg/µL; (**D**,**H**)). At 3 days after injection, animals were transcardially perfused and brain tissues were processed for OX-42 immunostaining (**A**–**D**) or OX-6 (**E**–**H**) immunostaining at 3 days post LPS. (**I**) Quantification of OX-42 process length. *** *p* < 0.001, significantly different from PBS (control). ^###^ *p* < 0.001, significantly different from LPS. Mean ± SEM; *n* of animals = 4 to 5 in each group. ANOVA and Bonferroni analysis. (**J**) Number of OX-6^+^ cells in the LPS-injected striatum (total area = 4.6 × 10^5^ μm^2^). *** *p* < 0.001 significantly different from PBS (control), ^#^ *p* < 0.01 significantly different from LPS mean ± SEM; *n* of animals = 4 to 5 in each group. ANOVA and Bonferroni analysis. ^#^ *p* < 0.05, significantly different from LPS. ^###^ *p* < 0.001, significantly different from LPS+IgG. ^$$^ *p* < 0.01, significantly different from LPS. ^$$$^ *p* < 0.001, significantly different from LPS.

To explore the potential function of endogenous IL-4 on striatal neurons, LPS was unilaterally co-injected with IL-4 neutralizing antibody (IL-4NA) to block the function of IL-4 into the striatum. Seven days later, similar to those shown in Figure 1, immunohistochemical analysis revealed a significant loss of Nissl^+^/NeuN^+^ cells in the LPS-injected striatum (Figure 3B,E), compared to the PBS-injected control (Figure 3A,E). By contrast, treatment with IL-4NA prevented the LPS-induced degeneration of Nissl^+^/NeuN^+^ cells in the striatum (Figure 3D,E), compared to the LPS-injected, IgG-treated striatum as a control (Figure 3C,E). Treatment with non-specific IgG alone had no effects on NeuN^+^/Nissl^+^ cells in the striatum (data not shown). Our recent work demonstrated no neurotoxic action of IL-4NA in a hippocampus treated with prothrombin kringle-2(pKr-2)+/IL-4NA+ and IL-4NA only (pKr-2-/IL-4NA+) [29]. In addition, the present results (Figure 3 and Figure 4) show a lack of neurotoxicity in the group of LPS−/IgG+, which might be comparable to those treated with LPS-/IL4-NA+. It is, therefore, likely that IL-4NA seems to be not toxic in the LPS-treated striatum, although we did not provide direct evidence in the striatum treated with LPS-/IL-4NA+.

IL-4 regulates activation of microglia in the LPS-injected substantia nigra [25] and prothrombin kringle-2-treated CA1 layer of the hippocampus [30]. Accordingly, we examined whether IL-4 could activate microglia in the LPS-injected striatum in vivo. Three days later, immunohistochemical analysis demonstrated that LPS produced microglial activation with a significant decrease in the OX-42^+^ cell process length (Figure 4B,I) in the striatum, compared to the PBS-injected control (Figure 4A,I). By contrast, treatment with IL-4NA prevented LPS-induced microglial activation, as determined by the OX-42^+^ cell process length in the striatum (Figure 4D,I), compared to the LPS-injected, IgG-treated striatum as a control (Figure 4C,I). Similar to OX-42^+^ cells, LPS increased the number of OX-6^+^ cells in the striatum (Figure 4F,J), compared to the PBS-injected control (Figure 4E,J). However, treatment with IL-4NA attenuated LPS-induced increases in the number of OX-6^+^ cells in the striatum (Figure 4H,J), compared to the LPS-injected, IgG-treated striatum as a control (Figure 4G,J). Treatment with non-specific IgG alone had no effects on microglia/macrophage cells in the striatum (data not shown).

### 2.2. Interleukin-4 Is Involved in Oxidative/Nitrosative Stress in LPS-Injected Rat Striatum In Vivo

**Figure 5:**
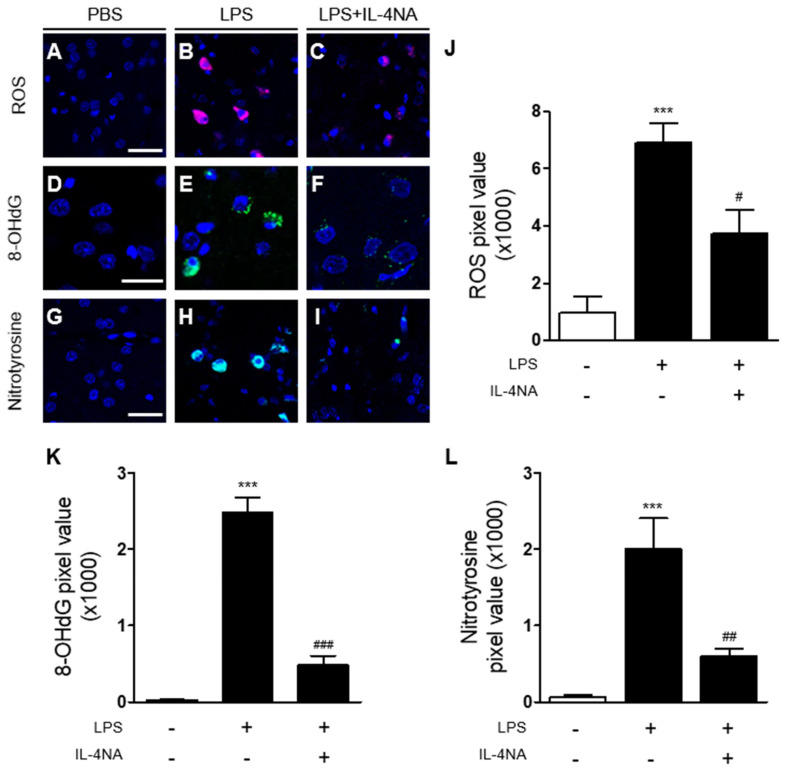
IL-4 induces oxidative/nitrosative stress in the LPS-injected striatum in vivo. Sections adjacent to those used in Figure 4 were prepared for immunohistochemical staining or hydroethidine histochemistry. (**A**–**C**) Hydroethidine histochemistry to detect oxidant production (ethidium fluorescence, red) in the striatum. Nuclei were counterstained with DAPI (blue). Scale bar, 30 µm. (**D**–**F**) Immunofluorescence images of 8-OHdG (green) to detect oxidative DNA damage in the striatum. Scale bar, 20 µm. Nuclei were counterstained with DAPI (blue). (**G**–**I**) Immunofluorescence images of nitrotyrosine (green) to detect nitrosative damage in the striatum. Nuclei were counterstained with DAPI (blue). Scale bar, 30 µm. (**J**) Quantification of ROS expression *** *p* < 0.001, significantly different from PBS. ^#^ *p* < 0.05, significantly different from LPS. Mean ± SEM; n of animals = 4 in each group. ANOVA and Bonferroni analysis. (**K**) Quantification of 8-OHdG expression *** *p* < 0.001, significantly different from PBS. ^###^ *p* < 0.001, significantly different from LPS. Mean ± SEM; n = 4 to 5 in each group. ANOVA and Bonferroni analysis. (**L**) Quantification of nitrotyrosine expression. *** *p* < 0.001, significantly different from PBS. ^##^ *p* < 0.01, significantly different from LPS. Mean ± SEM; n of animals = 5 in each group. ANOVA and Bonferroni analysis.

As IL-4 is associated with oxidative stress in the prothrombin kringle-2-injected hippocampus [29] and cortex [30], we hypothesized that IL-4 could induce oxidative/nitrosative stress in the LPS-treated striatum in vivo. To test this, we examined whether IL-4NA altered the effects of LPS on oxidative/nitrosative stress by analyzing levels of reactive oxygen species (ROS) (O_2_^−^) production, DNA oxidation, and protein nitration at 3 days post LPS. Hydroethidine histochemical analysis demonstrated that LPS injection increased O_2_^−^ production in the striatum in vivo (Figure 5B,J), compared to the PBS-injected control (Figure 5A,J). By contrast, treatment with IL-4NA significantly reduced LPS-induced O_2_^−^ production in the striatum (Figure 5C,J). For DNA oxidation, 8-OHdG levels were measured in the LPS-injected striatum in the presence or absence of IL-4NA. Immunohistochemical analysis showed that LPS injection increased the levels of 8-OHdG in the striatum (Figure 5E,K), compared to the PBS-injected control (Figure 5D,K). Treatment with IL-4NA partially mitigated the LPS-induced levels of 8-OHdG (Figure 5F,K). For protein nitration, analysis by nitrotyrosine revealed that LPS injection increased protein nitration (Figure 5H,L), compared with the PBS-injected control (Figure 5G,L). Treatment with IL-4NA attenuated the increase in levels of nitrotyrosine due to LPS injection (Figure 5I,L).

### 2.3. Interleukin-4 Regulates the M1 and M2 Activation State of Microglia/Macrophages in LPS-Injected Striatum In Vivo

**Figure 6:**
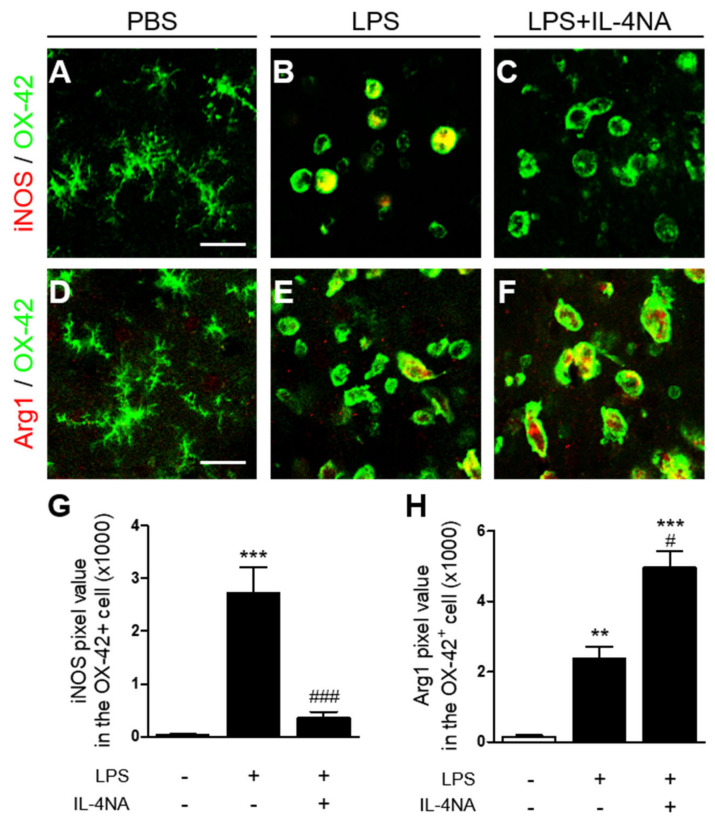
IL-4 modulates M1/M2 activation state of microglia/macrophages in LPS-injected striatum in vivo. Sections adjacent to those used in Figure 4 were prepared for immunohistochemical staining. (**A**–**C**) Immunofluorescence images of iNOS (inducible nitric oxide synthase, red) to identify classical activation of microglia/macrophages (M1 state) and OX-42 (green), and both images were merged (yellow) in striatum treated with PBS (**A**), LPS (**B**), and LPS and IL-4 NA (**C**). Scale bar, 25 µm. (**D**–**F**) Immunofluorescence images of Arg1 (arginase 1, red) to identify alternative activation of microglia/macrophages (M2 state) and OX-42 (green), and both images were merged (yellow) in striatum treated with PBS (**D**), LPS (**E**), and LPS and IL-4 NA (**F**). Scale bar, 25 µm. (**G**) Quantification of iNOS expression in OX-42^+^ cells. ** *p* < 0.01, *** *p* < 0.001, significantly different from PBS. ^###^ *p* < 0.001, significantly different from LPS. Mean ± SEM; n of animals = 4 to 5 in each group. ANOVA and Bonferroni analysis. (**H**) Quantification of Arg1 expression in OX-42^+^ cells. ** *p* < 0.01, *** *p* < 0.001 significantly different from PBS. ^#^ *p* < 0.05, significantly different from LPS. Mean ± SEM; n of animals = 4 in each group. ANOVA and Bonferroni analysis.

LPS induces microglial activation and microglial polarization in the substantia nigra [32]. Accordingly, we investigated whether LPS could induce microglia/macrophage polarization in the stratum in vivo by analyzing expression of iNOS and arginase 1 as an M1 and M2 marker, respectively. Immunohistochemical analysis revealed that iNOS expression in OX-42^+^ microglia/macrophages increased 25-fold in the LPS-injected striatum (Figure 6B,G, *p* < 0.001), compared to the PBS-injected control (Figure 6A,G). Expression of arginase 1 in OX-42^+^ microglia/macrophages increased 20-fold in the LPS-injected striatum (Figure 6E,H, *p* < 0.01), compared to the PBS-injected control (Figure 6D,H). Next, we examined whether IL-4 could influence LPS-induced polarization of microglia/macrophages in the striatum, resulting in neurodegeneration (Figure 3) and microglia/macrophage activation (Figure 4). Functional inhibition of IL-4 by IL-4NA significantly attenuated expression of iNOS on OX-42^+^ microglia/macrophages in the LPS-injected striatum (Figure 6C,G, *p* < 0.001), compared to LPS alone (Figure 6B,G). By contrast, IL-4NA treatment significantly increased arginase 1 expression on OX-42^+^ microglia/macrophages in the LPS-injected striatum (Figure 6F,H, *p* < 0.001), compared to LPS alone (Figure 6E,H). Taken together, these results suggest that IL-4 regulates LPS-induced polarization of microglia/macrophages by shifting from the M2 to the M1 state, resulting in neurodegeneration.

## 3. Discussion

Microglia, resident immune cells of the CNS, are associated with a variety of neuropathologies [33,34]. As reactive microglia/macrophages can express diverse surface receptors, such as complement receptors (OX-42) and the major histocompatibility complex (OX-6) [32,35], they are visualized and assessed by OX-42^+^ and/or OX-6^+^ cells [29]. When activated, OX-42^+^ cells exhibit a gradual change in morphology from a quiescent ramified form (resting state) to an amoeboid form (activated state) [36]. In this context, the present study demonstrated that LPS increased levels of amoeboid OX-42^+^ cells and OX-6^+^ cells in the striatum, compared to the control, indicating the activation of microglia/macrophages. This is consistent with findings showing that administration of LPS into the rat striatum led to localized activation of microglia/macrophages in the striatum, as evidenced by increases in OX-42^+^ or OX-6^+^ cells [37,38].

Microglia/macrophages become polarized upon various insults, including LPS, and are classified as M1 or M2 phenotypes [13,39]. The M1 microglia is found to produce proinflammatory cytokines, such as TNF, IL-6, IL-1β, and iNOS [32,35]. The M2 microglia express cytokines and receptors, such as IL-10, Ym1, Arg1, and ornithine that restore homeostasis [40,41]. Regarding this, intranigral injection of LPS produced the death of dopamine neurons in the substantia nigra with expression of both the M1 activation-state marker (iNOS, IL-1β, IL-6, and CD16) and the M2 activation-state marker (Arg1 and CD206) [32,42]. This study also demonstrated that treatment with capsaicin or ginsenoside Rg1 contributed to the survival of nigra dopamine neurons by reducing levels of iNOS, IL-1β, IL-6, and CD 16, and increasing levels of Arg1 and CD206, indicating a shift in the proinflammatory M1 microglia/macrophage population to an anti-inflammatory M2 state. Similarly, the present study showed that IL-4NA attenuated levels of iNOS expression and upregulated levels of Arg1 expression in OX-42^+^ microglia/macrophages in the LPS-injected striatum in vivo, subsequently rescuing striatal neurons. Thus, it is likely that IL-4 is neurotoxic via modulation of M1/M2 microglia/macrophage polarization in the LPS-injected striatum in vivo.

Reactive microglia/macrophages contribute to oxidative/nitrosative stress by producing ROS/RNS and oxidative damage to protein, lipid, and DNA [4,33], resulting in neurodegeneration in vivo [5,6]. Much experimental evidence, including ours, demonstrated that neurotoxicity induced by various insults, such as LPS, thrombin, pKr-2, and beta-amyloid_1-42_ was accompanied by reactive microglia/macrophages, increased levels of ROS/RNS, and oxidative damage to proteins, lipid, and DNA in the substantia nigra, hippocampus, and cerebral cortex [24,30,43,44]. These studies also showed that endogenous IL-4 was expressed exclusively in reactive microglia/macrophages, and neutralization of IL-4 by IL-4 neutralizing antibody (IL-NA) significantly increased neuronal survival. Accompanying neuroprotection, inhibition of IL-4 function by IL-4NA prevented activation of microglia/macrophages, and attenuated reactive oxygen species (ROS)-derived oxidative damage and nitrosative damage, as analyzed by immunohistochemistry and hydroethidine histochemistry. This is in line with our present findings showing that IL-4NA significantly increased survival of striatal neurons, inhibited activation of microglia/macrophages, and attenuated ROS/RNS production and oxidative damage. It is, therefore, likely that endogenous IL-4 expressed on reactive microglia/macrophages was neurotoxic in the LPS-injected striatum in vivo by activating microglia/macrophages and producing oxidative/nitrosative stress.

## 4. Materials and Methods

### 4.1. Animal and Stereotaxic Surgery

All experiments were conducted in accordance with approved protocols and guidelines established by the Committee on Animal Research of Kyung Hee University (KHSASP-21-364). Female Sprague–Dawley (SD) rats (240–270 g) were purchased from Daehan Biolink (introduced from Taconic Co., Petersburgh, NY, USA). Stereotaxic surgery was performed as previously described [45]. SD rats were anesthetized by injection of chloral hydrate (360 mg/kg, intraperitoneally, Sigma-Aldrich, St. Louis, MO, USA) and positioned in a stereotaxic apparatus (David Kopf Instruments, Tujunga, CA, USA). All injections were performed using a Hamilton syringe pump (700 series, Hamilton Company, Reno, NV, USA) with a 30S gauge, small-hub RN needle (point style 4, 1-inch needle length, 45′ tip angle, Hamilton) and equipped with an infusion/withdrawal syringe pump (KD scientific). According to the atlas of Paxinos and Watson (Paxinos, 1998, The Rat Brain in Stereotaxic Coordinates, 6th Edition), animals received a unilateral administration of LPS (right striatum; A/P 0.7, M/L -2.8, D/V -5.0; LPS, 5 µg in 3 µL phosphate-buffered saline, 1 µL/min, *Salmonella enteritidis*, Sigma-Aldrich). For IL-4 neutralization, IL-4 neutralizing antibody (1 µg/µL, R&D Systems, Minneapolis, MN, USA) or nonspecific IgG (1 µg/µL, R&D Systems) as a control, together with LPS, were injected into the equivalent coordinate of the right striatum. After injection, the needle was left in place for an additional 5 min before slow retraction. Intact (nontreated) or PBS-treated animals were used as controls. Animals were killed by an overdose of chloral hydrate for further study.

### 4.2. Immunohistochemistry and Immunofluorescence Double Labeling

Animals were transcardially perfused with a saline solution containing 0.5% sodium nitrate and heparin (10 U/mL), and then fixed with 4% paraformaldehyde dissolved in 0.1 M phosphate buffer (PB). Brains were removed from the skulls, postfixed overnight in buffered 4% paraformaldehyde at 4 °C, stored in a 30% sucrose solution for 24–48 h at 4 °C until they sank, and were frozen-sectioned on a sliding microtome in 40-µm-thick coronal sections. All sections were collected in six separate series and processed for immunohistochemical staining, as described previously [31]. In brief, brain sections were rinsed in PBS and then incubated overnight at room temperature with the following primary antibodies.

Table 1 shows them: mouse anti-OX-42 (1:400, Bio-Rad, Hercules, CA, USA), mouse anti-OX-6 (1:400, BD Biosciences, San Jose, CA, USA), mouse anti-neuron-specific nuclear protein (NeuN; 1:400, Merck Millipore, Darmstadt, Germany), mouse-anti-glial fibrillary acidic protein (GFAP; 1:500, Sigma), anti-8-hydroxy-2-deoxy guanosine (8-OHdG; 1:300, JaICA, Tokyo, Japan) for detecting oxidative DNA damage, mouse anti-nitrotyrosine (NT; 1:50, Abcam, Cambridge, MA, USA) for recognizing ROS/RNS-dependent protein damage, mouse rabbit-anti-inducible nitric oxide (iNOS; 1:200, BD Biosciences), anti-arginase 1 (1:200; Santa Cruz Biotechnology, Dallas, TX, USA), and goat-anti-interleukin-4 (IL-4; 1:400, R&D Systems, Minneapolis, MN, USA). The following day, brain sections were rinsed with PBS and 0.5% bovine serum albumin (BSA), and incubated with the appropriate.

Secondary antibodies processed with an avidin-biotin complex kit (Vector Laboratories, Burlingame, CA, USA). The bound antiserum was visualized by incubating it with 0.05% diaminobenzidine-HCl (DAB; Sigma) and 0.003% hydrogen peroxide in 0.1 M PB. The DAB reaction was stopped by rinsing tissues in 0.1 M PB. Labeled tissue sections were then mounted on gelatin-coated slides and analyzed under a bright-field microscope (Olympus Optical, Tokyo, Japan). For Nissl staining, some of the striatal tissues were mounted on gelatin-coated slides, dried for 1 hour at room temperature, stained with 0.5% cresyl violet acetate (Sigma), dehydrated, coverslipped, and then analyzed under a bright-field microscope (Olympus Optical, Tokyo, Japan).

For immunofluorescence double labeling, tissue sections were processed as described previously [31]. Briefly, 40-um-thick coronal sections were mounted on gelatin-coated slides, derided for 20 min at room temperature, rinsed in PBS, and incubated overnight at room temperature with primary antibodies, as used above. The following day, tissue sections were incubated with fluorescence-conjugated secondary antibodies (FITC-conjugated anti-mouse (1:500, Merck Millipore)), Cy3-conjugated anti-rabbit (1:1000, Merck Millipore), Alexa Fluor 594-conjugated anti-goat IgG (1:1000, Invitrogen, Waltham, MA, USA), and antibodies in 0.5% bovine albumin serum in PBS. Tissue sections were coverslipped with VECTASHIELD medium (Vector Laboratories), and viewed using confocal microscopy (LSM700, Carl Zeiss, Germany).

### 4.3. In Situ Detection of O_2_^−^ and O_2_^−^-Derived Oxidants

For in situ visualization of the O_2_^−^ and O_2_^−^ derived oxidants, hydroethidine histochemistry was carried out at 3 days post LPS. Animals intraperitoneally received hydroethidine (1 mg/kg in PBS containing 1% dimethyl sulfoxide; Sigma) and were transcardially perfused with a saline solution containing 0.5% sodium nitrate and heparin (10 U/mL), and then fixed with 4% paraformaldehyde in 0.1 M phosphate buffer. After fixation, the brain tissues were cut into 40-um slices using a sliding microtome. As described [30,46], tissue sections were mounted on gelatin-coated slides, and the oxidized hydroethidine product, ethidium, was viewed by confocal microscope (Carl Zeiss), and then merged with DAPI solution (Vector Laboratories). To quantify, obtained images were analyzed by Image J (National Institutes of Health, Bethesda, MD, USA).

### 4.4. Striatal Cell Counting 

As previously described in the SN immune cell [32], 40 μm coronal sections collected in six separate series were chosen and 4 evenly spaced sections were selected from anterior to posterior throughout the striatum. Quantification of cell number and intensity of immunoreactivity were performed using Adobe Photoshop CS6. Every selected section passed through the striatum region, containing up to 4.6 × 10^5^ μm^2^ of the striatum for DAB staining. Immunopositive cells were analyzed by the “count tool” under the Analysis menu.

### 4.5. Microglia Process Length Quantification

As described [47], a ramified cell has complicated processes originated from the cell soma. In the present study, LPS or LPS+IL-4NA induced changes in microglial ramification, which indicated a microglial response to an altered physiological status. All bright-field microscope images were converted into binary and skeletonized ones followed by the use of Image J software and appropriate plugins. Additionally, cell somas were manually counted for each bright-field microscope image. The AnalyzeSkeleton plugin was then applied to the skeleton image, which tags skeletal features relevant to microglia ramification. The number of microglia process lengths/cell was analyzed by summarizing the number of process lengths from the AnalyzeSkeleton plugin data output and all data were normalized by the number of microglia cell somas in each image.

### 4.6. Image J Analysis

As described [31], imaging data were analyzed in Image J (National Institutes of Health, USA) to quantify the expression of IL-4, nitrotyrosine, 8-OHdG, iNOS, and Arg1 in the LPS-injected striatum. Immunofluorescence was quantified by Image J with the colocalization plugin. Chromogenic signal intensity on the image obtained from the same area in each tissue sample was quantified by Image J with the color deconvolution plugin.

### 4.7. Statistics

All values are expressed as mean ± SEM. Values *p* < 0.05 were considered statistically significant and assessed by ANOVA with Newman–Keuls analysis or Bonferroni analysis (GraphPad Software, San Diego, CA, USA).

## 5. Conclusions

In summary, the current study demonstrates that intrastriatal injection of LPS causes death of neuronal cells and activation of microglial/macrophages in the striatum in vivo. This LPS-induced neurotoxicity was accompanied by significant production of ROS/RNS and cytokines of M1/M2 state microglia/macrophages, with IL-4 expression in the striatum exclusively within reactive microglia/macrophages. Treatment with IL-4 neutralizing antibodies protected striatal neurons against LPS-induced neurotoxicity in the striatum by inhibiting reactive microglia/macrophage-derived ROS/RNS production and oxidative damage, and attenuating iNOS expression and enhancing Arg1 expression. Collectively, our work suggests that IL-4 might regulate expression of M1- and M2-related factors such as pro- and anti-inflammatory cytokines and ROS/RNS by regulating the polarized state of microglia/macrophages in the LPS-injected striatum, resulting in degeneration of striatal neurons.

## Figures and Tables

**Table 1:** Primary antibodies used for IHC and IF.

Primary Antibody	Dilution	Company	Catalog No.
OX-42	1:400	Bio-Rad	MCA275G
OX-6	1:400	BD Biosciences	554926
NeuN	1:1000	Merck	MAB377
GFAP	1:500	Sigma-Aldrich	G3893
IL-4	1:400	R&D Systems	AF504
iNOS	1:200	BD Biosciences	610333
Arg1	1:200	Santa Cruz	SC-166920
8-OHdG	1:300	JaICA	MOG-100P
Nitrotyrosine	1:50	Abcam	ab7048
